# Prevalence of small intestinal bacterial overgrowth in intestinal failure syndrome: A systematic review and meta‐analysis

**DOI:** 10.1111/jgh.16668

**Published:** 2024-06-27

**Authors:** Ayesha Shah, Thomas Fairlie, Mark Morrison, Neal Martin, Karin Hammer, Johann Hammer, Natasha Koloski, Ali Rezaie, Mark Pimentel, Purna Kashyap, Michael P Jones, Gerald Holtmann

**Affiliations:** ^1^ Faculty of Medicine University of Queensland Brisbane Queensland Australia; ^2^ Department of Gastroenterology and Hepatology Princess Alexandra Hospital Brisbane Queensland Australia; ^3^ Translational Research Institute Brisbane Queensland Australia; ^4^ Faculty of Medicine, Frazer Institute University of Queensland Woolloongabba Queensland Australia; ^5^ St. Anna Kinderspital Medical University of Vienna Vienna Austria; ^6^ Department of Gastroenterology and Hepatology Medical University of Vienna Vienna Austria; ^7^ Karsh Division of Gastroenterology and Hepatology, Department of Medicine, Medically Associated Science and Technology (MAST) Program Cedars‐Sinai Los Angeles California USA; ^8^ Division of Gastroenterology and Hepatology Mayo Clinic Rochester Minnesota USA; ^9^ School of Psychological Sciences Macquarie University Sydney New South Wales Australia

**Keywords:** bacterial overgrowth, breath tests, intestinal failure, prevalence, short gut syndrome, SIBO

## Abstract

**Background and Aim:**

Patients with intestinal failure (IF) have abnormal intestinal anatomy, secretion, and dysmotility, which impairs intestinal homeostatic mechanisms and may lead to small intestinal bacterial overgrowth (SIBO). We conducted a systematic review and meta‐analysis to determine the prevalence of SIBO in patients with IF and to identify risk factors for SIBO.

**Methods:**

MEDLINE (PubMed) and Embase electronic databases were searched from inception to December 2023 for studies that reported the prevalence of SIBO in IF. The prevalence rates, odds ratio (OR), and 95% confidence intervals of SIBO in IF and the risk factors for SIBO in IF were calculated using random effects model.

**Results:**

Final dataset included nine studies reporting on 407 patients with IF. The prevalence of SIBO in IF was 57.5% (95% CI 44.6–69.4), with substantial heterogeneity in this analysis (*I* ^2^ = 80.9, *P* = 0.0001). SIBO prevalence was sixfold higher in patients with IF who received parenteral nutrition (PN) compared with IF patients not on PN (OR = 6.0, 95% CI 3.0–11.9, *P* = 0.0001). Overall, the prevalence of SIBO in patients with IF using PPI/acid‐suppressing agents (72.0%, 95% CI 57.5–83.8) was numerically higher compared with IF patients not using these agents (47.6%, 95% CI 25.7–70.2).

**Conclusions:**

This systematic review and meta‐analysis suggests that there is an increased risk of SIBO in patients with IF and that PN, and potentially, the use of PPI/acid‐suppressing agents is risk factors for SIBO development in patients with IF. However, the quality of evidence is low and can be attributed to lack of case–control studies and clinical heterogeneity seen in the studies.

## Introduction

Intestinal failure (IF) is defined as the impairment of gut function below the minimum necessary for the absorption of macronutrients and/or water and electrolytes, such that intravenous supplementation is required to maintain health and/or growth.^1^ The underlying pathophysiology of IF can be grouped into five categories, including short bowel, intestinal fistula, intestinal dysmotility, mechanical obstruction, and mucosal disease.^2^ Short bowel syndrome/short gut syndrome is the most common cause of intestinal failure in adults and children.^3^ Parenteral nutrition (PN) and home PN remain the mainstay of therapy, independent of the nature of IF,^4^ which can be total or partial and permanent or temporary. The administration of PN requires a central venous catheter and hence increases the risk for catheter related blood stream infections (CRBSI).^5^

Patients with IF may have abnormal intestinal anatomy, secretion, and dysmotility, which impairs intestinal homeostatic mechanisms and may lead to small intestinal bacterial overgrowth (SIBO). Small intestinal bacterial overgrowth (SIBO) is one of the most widely recognized and studied manifestation of gut microbial dysbiosis.^6^ SIBO is a clinical disorder, characterized by a wide spectrum of gastrointestinal symptoms,^7^ which often overlap with other gastrointestinal conditions^8^ and may cause structural changes such as atrophy of small intestinal villi^9^ with subsequent alterations of small intestinal absorption. Historically, the presence of ≥ 10^5^ colony forming units per milliliter (CFU/mL) of colonic‐type bacteria in culture of jejunal aspirate has been considered the “gold standard” for establishing diagnosis of SIBO.^10^ However, more recent data identified ≥ 10^3^ CFU/mL of duodenal aspirate as the optimal threshold for diagnosing SIBO.^11^ Although duodenal aspirate and culture are considered the gold standard for diagnosing SIBO, breath tests are commonly used in clinical practice due to their simplicity. However, it is worth noting that breath tests have limited sensitivity and specificity when compared with culture.^10^

Several studies have reported that SIBO and CRBSI are two of the most common complications in patients with IF, with a direct impact both on morbidity and on mortality.^12^, ^13^ Furthermore, SIBO has been associated with an increased risk of CRBSI due to bacterial translocation, triggering intestinal mucosal inflammation, which may affect the initiation of enteral feeding and the transition of patients from PN.^13^, ^14^, ^15^ Thus, knowledge of the risk factors and their mitigation is critical to the long‐term health and well‐being of patients with IF. In that context, the use of antimicrobials is a common treatment approach for SIBO. While this treatment often leads to symptom improvement and normalization of a positive test for SIBO^16^ in other scenarios, SIBO often tends to be a recurrent problem in IF and empirical treatment strategies, such as intermittent or cycling antibiotic regimens may be chosen and monitored based on the patient's clinical response. However, there are concerns associated with this approach, including the potential misuse of antibiotics, antibiotic related adverse effects, and the development of drug resistance.

Against this background, we conducted a systematic review and meta‐analysis with a primary objective to determine the prevalence of SIBO in patients with IF. In addition, we aimed to explore (i) the association between PN (and complications of PN) and SIBO in patients with IF; (ii) the etiology of IF and SIBO; (iii) the effect of diagnostic modality and acid‐suppressing agents (e.g. proton pump inhibitors [PPIs] and histamine‐2 receptor antagonists [H2RAs]) use on the prevalence of SIBO in patients with IF; and (iv) the association between SIBO and anatomical changes in patients with IF. We also aimed to (v) assess the effect of antibiotic therapy on symptom improvement in IF patients with SIBO.

## Methods

### Protocol and registration

This systematic review and meta‐analysis meets the preferred reporting items for systematic reviews and meta‐analysis statement requirements (PRISMA).^17^, ^18^ The protocol for this Systematic Review was prospectively registered with PROSPERO (CRD42023414010).

### Search strategy

**Figure 1:**
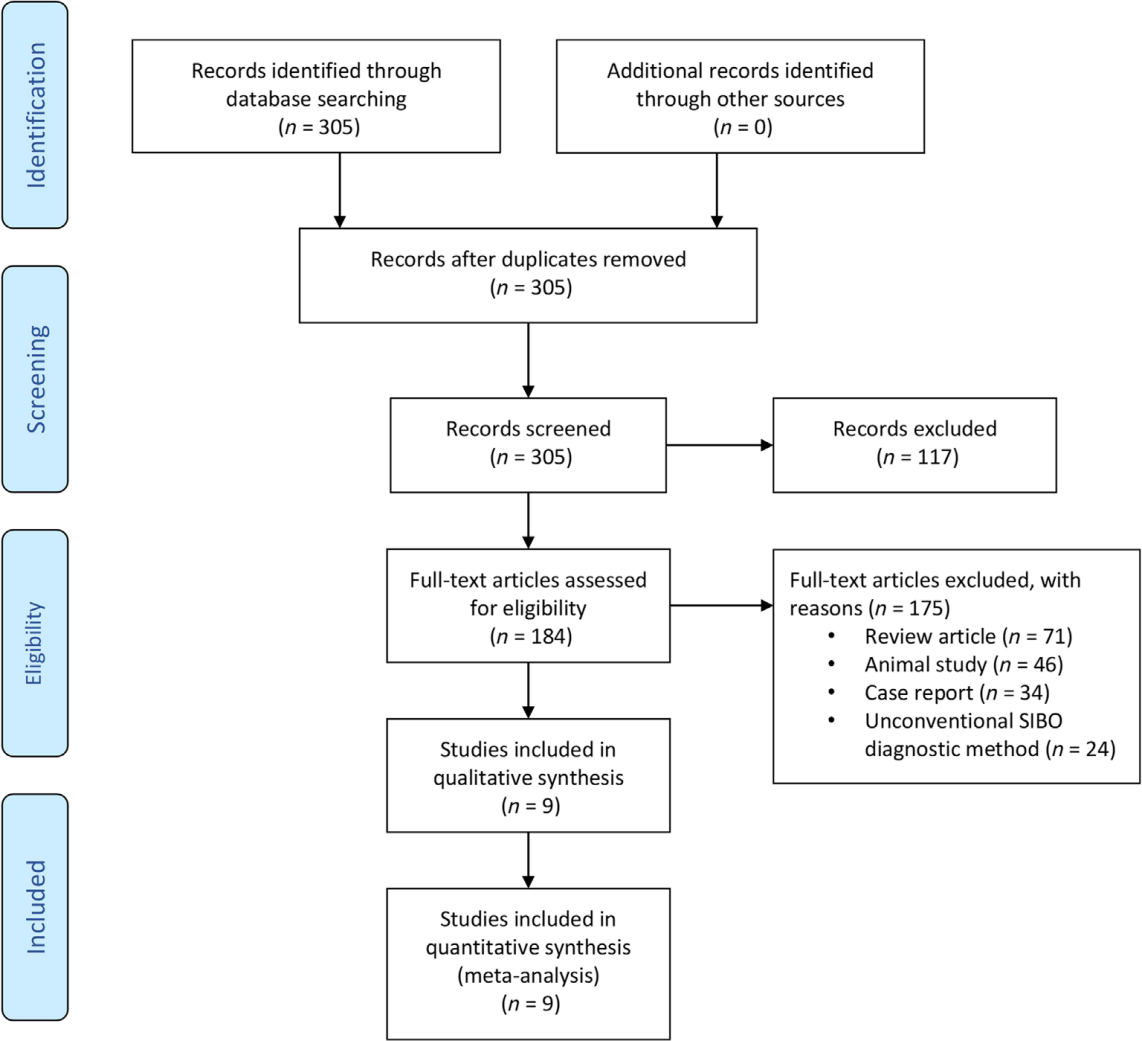
PRISMA flow diagram.

Electronic databases, including PUBMED, MEDLINE (OvidSP), and EMBASE, were searched from initiation (1966) up to December 2023 for all studies assessing the prevalence of SIBO in patients with IF and/or short gut syndrome (SGS)/short bowel syndrome (SBS). The literature search strategy is outlined in the PRISMA flow diagram (Fig. 1) and was conducted with the assistance of our librarian. The search strategy for MEDLINE has been outlined in Figure S1. The initial search was not limited to specific languages to capture all appropriate studies. A further advanced search was conducted. Gray literature was searched with Google and Google Scholar, and the “Snowball” method was also utilized to identify all relevant articles.

### Selection of studies

Two authors (A. S. and T. F.) independently screened abstracts and titles. Abstracts were eliminated if the study did not investigate the association between SIBO and IF or SBS. Full texts of the remaining articles were retrieved and reviewed. Case–control and cohort studies, recruiting unselected subjects with a confirmed diagnosis of IF that reported the prevalence of SIBO using clinically validated methods and/or gastrointestinal symptoms suspicious for SIBO in patients with IF, and compared the prevalence SIBO in IF *versus* controls were eligible for inclusion. We also included studies that reported on efficacy data after antibiotic treatment of SIBO in patients with IF. If a study was interventional, both intervention and control groups were included. If a study was longitudinal or interventional, only the baseline data were used. The diagnosis of IF was based upon the clinical assessment by the treating physician. Studies not reporting original data, case reports or case series, animal studies, review articles, and those not providing data on SIBO in IF were excluded. Conference abstracts that provided available data were also included in the study. Individuals in the control group included healthy asymptomatic controls as well as “patient controls” including patients undergoing evaluation for unexplained “gastrointestinal syndromes” (e.g. iron deficiency anemia and dysphagia).

Eligibility criteria for study inclusion are provided in Table S1. Disagreements between reviewers were resolved by mutual consensus after reference to the original published paper.

### Data extraction and quality assessment

Data were extracted independently by two authors with discrepancies resolved by reference to the source publication. Data were entered into a Microsoft Excel spreadsheet (2016 Professional edition: Microsoft Corp, Redmond, Washington, USA). The following information was extracted from each study independently by the two reviewers (A. S. and T. F.): author, year of publication, journal, study design, country, source of controls, method of diagnosis of SIBO including test duration, quantity of substrate used and the cut off criteria for diagnosis of SIBO, mean age, gender, concurrent use of acid‐suppressing agents, antibiotics, fecal calprotectin, and previous gastrointestinal surgeries (including presence of ileo‐cecal valve, length [percentage] of residual small bowel/colon) for patients with IF. In addition, for all patients with IF, data regarding mode of diagnosis of IF, causes of IF, treatment with PN, duration of treatment with PN, episodes of CRBSI, gastrointestinal symptoms associated with SIBO, treatment of SIBO with antibiotics (type of antibiotics, dosing, timing, and duration of therapy), and objective and subjective response post treatment were recorded. The quality of the cohort studies included was assessed by using the Joanna Briggs Institute (JBI) critical appraisal tools for use in JBI systematic reviews for cohort studies.^19^ The risk of bias was ranked as high when the study reached up to 49% of “yes” score, moderate when the study reached from 50 to 69% of “yes” score, and low when the study reached over 70% of “yes” score.

### Data analysis

In an initial step, case numbers of patients with IF (using various modalities for SIBO diagnosis) were determined. Data were recorded as frequency (*N*) of patients in each category, along with means and SD for quantitative variables. The median value and interquartile range were transformed to means and SD.^20^ The interquartile range or the 5th and 95th percentile ranges were converted to SD through the following formula: SD = 0.7413 × (values at 75th percentile − values at 25th percentile) or SD = (values at 95th percentile − values at 5th percentile)/(2 × 1.645).^21^

In a second step, the pooled prevalence rates and 95% confidence intervals (CI) for the prevalence of SIBO in patients with IF were calculated. Subgroup analysis stratified by diagnostic modalities, etiology of IF, effect of PN and CRBSI, and effect of acid‐suppressing agents in patients with IF were carried out. Lastly, we calculated the efficacy of antibiotic treatment in SIBO positive patients with IF. We only summarized acid‐suppressing agents (including PPI use, antacids, and H2RAs)/antibiotics data that were reported in included case–control and cohort studies. In analysis with two studies included, pooled study means and standard deviations were calculated according to the Cochrane methodology, whereby the effect of SIBO diagnosis on PN duration and remnant small and long bowel was evaluated with two‐way ANOVA. Finally, we did sensitivity analysis including only high‐quality studies, reporting the prevalence of SIBO in patients with IF.

Analyses for the association between SIBO and IF were carried out utilizing the Comprehensive Meta‐Analysis Software (CMA) Version 3.3.070. NJ, USA. In the Results section, we report the observed (unweighted) number of positive cases and total tested in addition to the weighted pooled estimates. Odds ratio and pooled prevalence estimates of disease were calculated using a random effects model (DerSimmonian & Laird method)^22^ to appropriately account for between‐study variability. The statistical package CMA utilized a logit transformation of proportions and the variance of the logit to estimate pooled event rates within groups and to compare event rates between groups. If one or more cells had a value of 0, then the CMA software automatically adds a fixed value of 0.5 to the respective cell for computation of log odds ratio and variance. Between‐study variation was evaluated using Cochrane's test^23^ and was quantified through the *I* ^2^ index in which values close to 100 indicate substantial variation between studies while values close to zero indicate minimal between‐study variation. Standard approaches (Egger's test^24^ and inspection of funnel plots) were applied to identify potential publication biases, for analyses when at least 10 studies included in the meta‐analysis. Further, either Cochrane's test *P* < 0.10 or *I* ^2^ > 50% was taken as an indication of substantial heterogeneity.

## Results

### Selection outcome

The initial literature search revealed 305 publications. Of these, 184 published articles appeared to be relevant for the study question and were retrieved for further evaluation. Of these, 175 were excluded for reasons explained in Figure 1, leaving nine eligible studies (Fig. 1). Three out of the nine studies were case–control studies,^13^, ^25^, ^26^ and the remaining six were cohort studies.^14^, ^27^, ^28^, ^29^, ^30^, ^31^ In all three case–control studies,^13^, ^25^, ^26^ control subjects did not undergo assessment for SIBO; hence, only the cases are included for analyses in this meta‐analysis. All studies, except one^30^ assessed SIBO in patients with IF in a pediatric populations. Therefore, a subgroup analysis comparing SIBO prevalence in adults and children with IF could not be performed. The characteristics of all the studies in the current meta‐analysis including the methodology pertaining to diagnosis of SIBO, patient characteristics, and geographic region are outlined in Table 1, Table S2, and Table S3. The summary of findings is outlined in Table 3.

**Table 1:** Characteristics of studies showing mode of diagnosis and prevalence of SIBO in patients with intestinal failure (IF)

No.	Author	Study year	Region	Type of study	Patients with IF, *n*	IF categories, *n*		Mode of diagnosis of SIBO	SIBO in IF patients, *n* (%)
						SBS	Non‐SBS		
1	Lilja *et al*.^1^	2015	Sweden	Case–control^†^ Prospective	11	11	NA	Gastrointestinal symptoms suspicious for SIBO	4 (36.4)
2	Galloway *et al*.^2^	2019	USA	Case–control^†^ Prospective	14	NA	NA	Duodenal aspirate culture	6 (42.9)
3	Gutierrez *et al*.^3^	2006	USA	Cohort Retrospective	57	NA	NA	Duodenal aspirate culture	40 (70.2)
4	Cole *et al*.^4^	2010	USA	Case–control^†^ Prospective	10	10	NA	GHBT	5 (50)
5	Belza *et al*.^5^	2020	Canda	Cohort Retrospective	102	75	27	Gastrointestinal symptoms suspicious for SIBO	35 (34.3)
6	Culbreath *et al*.^6^	2022	USA	Cohort Retrospective	104	NA	NA	Duodenal aspirate culture	78 (75.0)
7	McGrath *et al*.^7^	2019	Australia	Cohort Retrospective	17	NA	NA	Small bowel aspirate culture OR HBT	12 (70.6)
8	Dibaise *et al*.^8^, ^‡^	2006	USA	Cohort Retrospective	43	43	NA	Duodenal aspirate culture OR GHBT	27 (62.8)
9	Kaufman *et al*.^9^	1997	USA	Cohort Retrospective	49	49	NA	Duodenojejunal aspirate culture	30 (61.2)

GHBT, glucose hydrogen breath test; n, number; NA, not available; SBS, short bowel syndrome; SIBO, small intestinal bacterial overgrowth.

^†^ Control subjects did not undergo assessment for SIBO; hence, only cases are included for analyses in this systematic review and meta‐analysis.

^‡^ Only study that included adult patients with intestinal failure.

### Prevalence of small intestinal bacterial overgrowth in patients with intestinal failure

**Figure 2:**
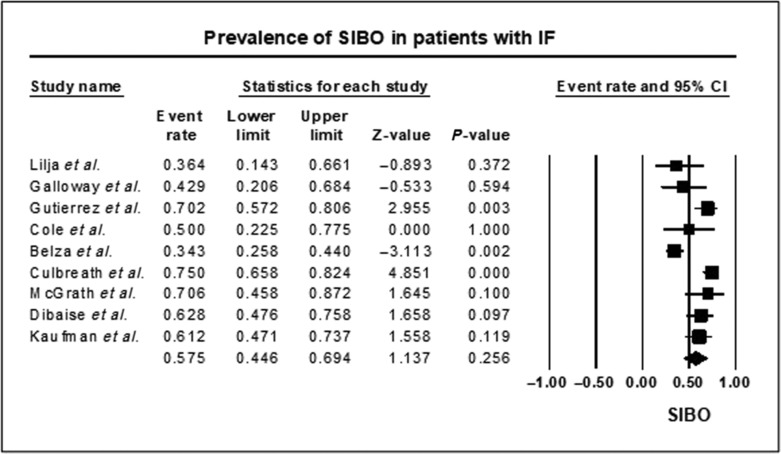
Forest plot of studies showing prevalence of SIBO in patients with IF. Overall, the prevalence of SIBO in patients with IF is 57.5% (95% CI 44.6–69.4) (*I* ^2^ = 80.9, *P* = 0.0001).

Nine studies^13^, ^14^, ^25^, ^26^, ^27^, ^28^, ^29^, ^30^, ^31^ reported the prevalence of SIBO in 407 patients with IF. Overall, the prevalence of SIBO in patients with IF was 57.5% (95% CI 44.6–69.4, Fig. 2), with substantial heterogeneity in the studies included in this analysis (*I* ^2^ = 80.9, *P* = 0.0001). However, none of the studies reported on the duration of IF and SIBO; hence, a subgroup analysis could not be performed.

In four studies,^14^, ^26^, ^27^, ^28^ SIBO was diagnosed using duodenal aspirate and culture using a cut‐off threshold of 10^5^ CFU/mL. Two studies^29^, ^30^ utilized either breath test or small bowel aspirate and culture, and in one study,^13^ the diagnosis was made using the glucose hydrogen breath test. However, in one study,^31^ the diagnosis of SIBO was based on clinical symptoms, and in another study,^25^ the diagnostic modality for SIBO was not specified (Table 1). As a result, it was not feasible to extract data for subgroup analysis to evaluate the impact of diagnostic modality on the prevalence of SIBO in patients with IF.

### Influence of risk of bias on the small intestinal bacterial overgrowth prevalence in patients with intestinal failure

#### High‐quality studies with low risk of bias

Utilizing the JBI critical appraisal tool (Table S4), out of the nine studies, four were classified as having a high risk of bias, four had a low risk of bias, and one study had a moderate risk of bias.

When only four high‐quality studies^14^, ^26^, ^27^, ^28^ were included, compared with controls, a higher prevalence of SIBO was found in patients with IF at 66.1% (95% CI 54.9–75.8, Fig. S4). There was moderate heterogeneity noted in the studies included in this analysis (*I* ^2^ = 58.2, *P* = 0.699).

### Prevalence of small intestinal bacterial overgrowth in patients with intestinal failure on parentral nutrition

**Figure 3:**
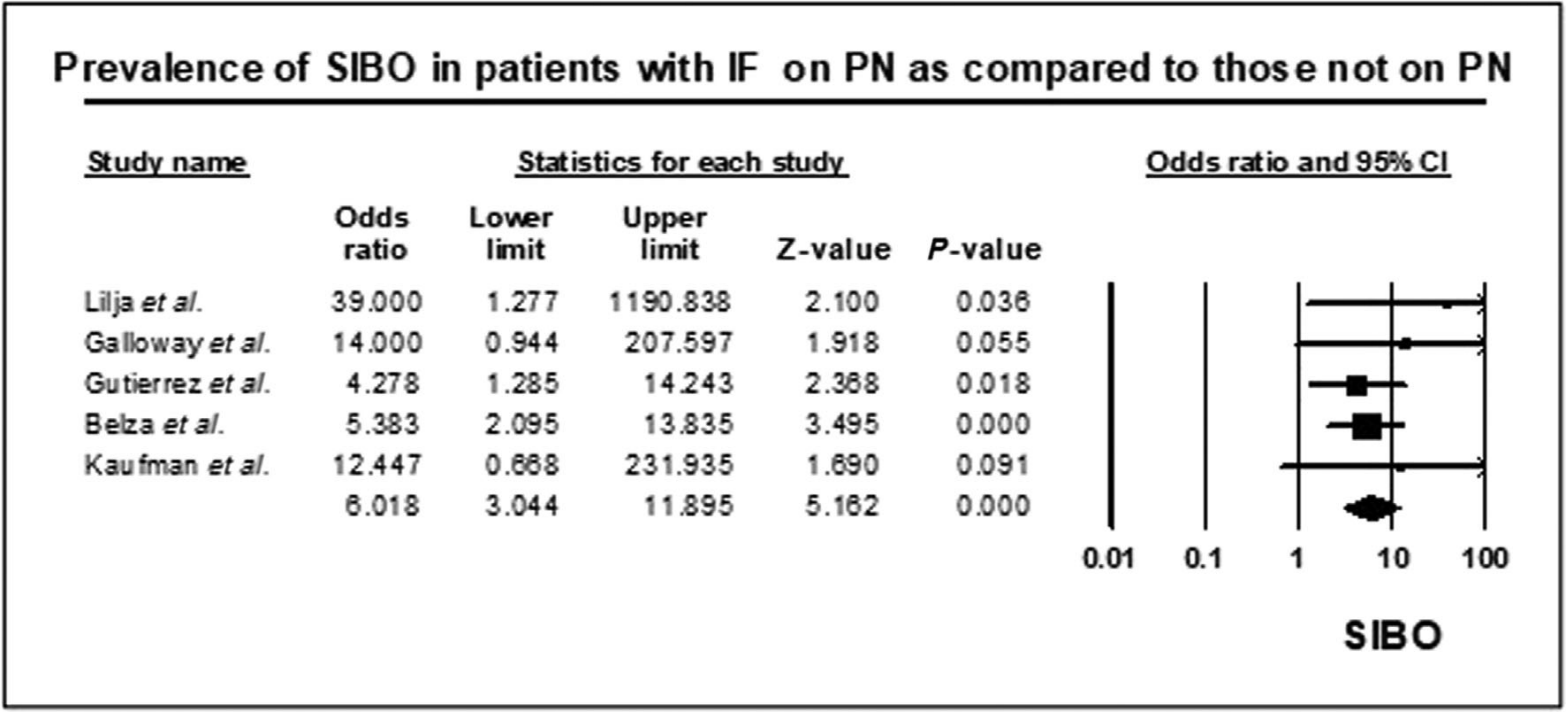
Forest plot of studies showing prevalence of SIBO in patients with IF, on parenteral nutrition (PN) (OR = 6.0 [95% CI 3.0–11.9], *P* = 0.0001) (*I* ^2^ = 0, *P* = 0.713).

Seven studies^13^, ^14^, ^25^, ^26^, ^27^, ^29^, ^31^ reported the prevalence of SIBO in 105 patients with IF patients who were on PN. Overall, the prevalence of SIBO in IF patients on PN was 74.9% (95% CI 59.8–85.6, Fig. S2). However, there was moderate heterogeneity in the analysis (*I* ^2^ = 35.5, *P* = 0.157). SIBO prevalence was sixfold higher in patients with IF who received PN compared with IF patients not on PN (OR = 6.0, 95% CI 3.0–11.9, *P* = 0.0001, Fig. 3). There was minimal heterogeneity in the studies included in this analysis (*I* ^2^ = 0, *P* = 0.713). Two studies^14^, ^27^ investigated the relationship between SIBO and the duration of PN in patients with IF. The duration of PN therapy was more than twice as long for SIBO positive patients with IF as compared with SIBO negative patients; however, it did not reach statistical significance (34.5 ± 41.5 months *vs* 13.9 ± 12.6 months *P* = 0.097).

### Link between small intestinal bacterial overgrowth and catheter related blood stream infections in patients with intestinal failure

Two studies^13^, ^27^ assessed the link between CRBSI and SIBO in patients with IF (Table 2). Overall, there was no difference in the prevalence of CRBSI in 15/33 IF patients with SIBO (45.5%, 95% CI 28.1–63.7) as compared with 5/11 IF patients without SIBO (45.5%, 95% CI 16.8–76.7).

**Table 2:** Characteristics of studies showing SIBO prevalence patients with intestinal failure (IF) on parenteral nutrition (PN) and associated complications

No.	Author	Age (years), mean (SD)	Gender (females), *n* (%)	Patients with IF, *n*	SIBO in patients with IF, *n* (%)	IF patients on PN, *n*	SIBO in IF patients on PN, *n* (%)	IF patients not on PN, *n*	SIBO in IF patients not on PN, *n* (%)	IF patients with CRBSI, *n*	SIBO in IF patients with CRBSI, *n* (%)
1	Lilja *et al*.^1^	3.5 (1.5)	NA	11	4 (36.4)	5	4 (80.0)	6	0	NA	NA
2	Galloway *et al*.^2^	5.8 (2.6)	NA	14	6 (42.9)	5	4 (80.0)	9	2 (22.2)	NA	NA
3	Gutierrez *et al*.^3^	5.7 (6.1)	30 (52.6)	57	40 (70.2)	34	28 (82.4)	23	12 (52.2)	12	10 (83.3)
4	Cole *et al*.^4^	0.8 (0.4)	4 (40)	10	5 (50)	10	5 (50.0)	NA	NA	8	5 (62.5)
5	Belza *et al*.^5^	NA	43 (42.2)	102	35 (34.3)	27	17 (63.0)	75	18 (24)	NA	NA
6	Culbreath *et al*.^6^	4.2 (2.73) *	25 (44.6) *	104	78 (75.0)	NA	44 (NA)	NA	12 (NA)	NA^†^	NA^†^
7	McGrath *et al*.^7^	NA	5 (29.4)	17	12 (70.6)	17	12 (70.6)	5	NA	NA	NA
8	Dibaise *et al*.^8^	48.1 (NA)	50 (57.5) ^#^	43	27 (62.8)	NA	22 (NA)	NA	5	NA	NA
9	Kaufman *et al*.^9^	5.9 (4.5)	NA	49	30 (61.2)	7	7 (100)	42	23 (54.8)	NA	NA

CRBSI, catheter related blood stream infections; n, number; NA, not available; SD, standard deviation.

^†^ Found no difference in the number of episodes of CRBSI (7 *vs* 8, *P* = 0.811) in the SIBO positive IF patients during the 6 months before and after antibiotic therapy.

**Table 3:** Summary of findings

SIBO in patients with IF	No. of studies	Patients with IF, *n*	SIBO in patients with IF, *n*	Prevalence of SIBO in IF patients, % (95% CI)	Assessment of heterogeneity between studies
All studies	9	407	237	57.5 (44.6–69.4)	*I* ^2^ = 80.9, *P* = 0.0001
High‐quality studies	4	224	154	66.1(54.9–75.8)	*I* ^2^ = 58.2, *P* = 0.699
Studies assessing SIBO in IF patients on PN	7	105	77	74.9(59.8–85.6)	*I* ^2^ = 35.5, *P* = 0.157
Studies assessing SIBO in IF patients due to short bowel syndrome	5	188	90	49.0(33.8–64.5)	*I* ^2^ = 73.4, *P* = 0.005

SIBO in patients with IF	No. of studies	Patients with IF, *n*	SIBO in patients with IF, *n*	SIBO in IF patients, OR (95% CI)	Assessment of heterogeneity between studies
Studies comparing SIBO in IF patients on PN compared with IF patients not on PN	5	IF patients on PN, *n* = 78 IF patients not on PN, *n* = 155	SIBO in IF patients on PN, *n* = 60 SIBO in IF patients not on PN, *n* = 55	6.0. 3.0–11.9, *P* = 0.0001	*I* ^2^ = 0, *P* = 0.713

CI, confidence interval; IF, intestinal failure; OR, odds ratio; PN, parenteral nutrition; PPI, proton pump inhibitor; SIBO, small intestinal bacterial overgrowth.

### Microbes cultured from small bowel aspirates in patients with intestinal failure diagnosed with small intestinal bacterial overgrowth

Three studies^26^, ^27^, ^28^ provided data on the microbes cultured from IF patients diagnosed with SIBO, table S6. In all three studies, the most common Gram‐positive bacteria were reported to be *Streptococcus viridans* and *Enterococcus* species, and the most common Gram‐negative bacteria were reported to be *Escherichia coli* and *Klebsiella pneumoniae*.

### Prevalence of small intestinal bacterial overgrowth, stratified according to etiology of intestinal failure

Five studies^13^, ^14^, ^25^, ^30^, ^31^ examined the prevalence of SIBO in patients categorized based on the etiology of IF, primarily distinguishing between those attributed to SBS and other non‐SBS causes. The pooled prevalence of SIBO in 188 patients with IF due to SBS was 49.0% (95% CI 33.8–64.5, fig. S3), with substantial heterogeneity in the analysis (*I* ^2^ = 73.4, *P* = 0.005). In one study^31^ that compared the prevalence of SIBO in IF patients with and without SBS, there was no significant differences between the two groups (32%, 95% CI 21.7–43.8 with SBS *vs* 40.7%, 95% CI 22.4–61.2 without SBS). In the remaining studies, the data could not be extracted for subgroup analysis.

### Effect of acid‐suppressive therapy on the prevalence of small intestinal bacterial overgrowth in intestinal failure

Only two studies^26^, ^27^, ^28^ reported on the prevalence of SIBO in 50 patients with IF who were on acid‐suppressing agents (Table S5). However, neither study provided information on the duration of PPI use among patients with IF. Overall, the prevalence of SIBO in patients with IF patients on acid‐suppressing agents (72.0%, 95% CI 57.5–83.8) was numerically higher compared with IF patients not on an acid‐suppressing agent (47.6%, 95% CI 25.7–70.2), but the difference was not statistically significant.

### Effect of antibiotic treatment on symptoms in patient with intestinal failure with small intestinal bacterial overgrowth

Although two studies^14^, ^31^ reported transient improvement in gastrointestinal symptoms in patients with IF with SIBO following treatment with antibiotic therapy, data could not be extracted to conduct subgroup analysis (Table S2).

### Link between small intestinal bacterial overgrowth and anatomical changes in patients with intestinal failure

#### Link between SIBO and ileocecal valve (ICV) in patients with IF

Four studies^25^, ^26^, ^27^, ^31^ assessed the link between an intact ileocecal valve (ICV) and SIBO in patients with IF (Table S8). Although the prevalence of SIBO in patients with IF without an ICV was higher compared with IF patients with an intact ICV (OR = 1.7, 95% CI 0.8–3.6, *P* = 0.174, Fig. S5), this failed statistical significance. There was minimal heterogeneity in the analysis (*I* ^2^ = 10.0, *P* = 0.343).

#### Link between SIBO and length of the remnant small bowel in patients with IF

Three studies investigated the relationship between SIBO and the percentage of remnant small bowel in patients with IF; however, data could be extracted from only two of those studies^13^, ^26^ (Table S9). We found that patients with IF and SIBO had approximately half of the remnant small bowel than those without SIBO; however, this failed to reach statistical significance (24.6% ± 13.9% *vs* 42.4% ± 30.5%. *P* = 0.089).

#### Link between SIBO and length of the remnant colon in patients with IF

Three studies investigated the relationship between SIBO and the percentage of remnant large bowel in patients with IF; however, data could be extracted only from two studies^13^, ^25^ (Table S10). We found that patients with IF and SIBO had similar remnant colon than those without SIBO (62.6% ± 25.1% *vs* 76.4% ± 25.1%, *P* = 0.21).

#### Link between SIBO in patients with IF and gastrointestinal symptoms

Two studies^28^, ^29^ investigated the predominant symptoms in patients with IF diagnosed with SIBO. While McGrath *et al*.^29^ found diarrhea to be the predominant symptoms among these patients, Culbreath et al.^28^ found emesis and feeding intolerance, high stool output, and abdominal distention as the predominant symptoms associated with SIBO in patients with IF. However, data could not be extracted to conduct subgroup analysis (Table S8).

## Discussion

To the best of our knowledge, this is the first systematic review and meta‐analysis reporting the prevalence of SIBO in patients with IF. This meta‐analysis included nine studies conducted in four countries, with 407 patients with IF. Overall, the data suggest an increased prevalence of SIBO in patients with IF [57.5% (95% CI 44.6–69.4)]. PN was a strong risk factor for SIBO in patients with IF (OR = 6.0, 95% CI 3.0–11.9, *P* = 0.0001). Furthermore, patients with SIBO and IF required PN for twice as long as compared with patients with IF without SIBO, although due to small sample size this difference did not reach statistical significance.

We found statistically significant heterogeneity in the primary analysis and moderate to substantial heterogeneity in majority of the subgroup analyses. To explore this heterogeneity, we did an additional sensitivity analysis, by including only “high‐quality” studies based upon JBI appraisal tool; however, this did not significantly reduce the heterogeneity. Given this, these high heterogeneity scores likely can be attributed to the inherent limitation of the studies included in this systematic review and meta‐analysis. Although three of the nine studies included in this meta‐analysis were case–control studies, the control subjects did not undergo testing for SIBO, hence were excluded from the analysis. Majority of the studies (six of the nine studies) were retrospective audits of insufficiently defined study cohorts, with limited information regarding etiology of IF or potential confounders (e.g., acid‐suppressing agents, previous antibiotic therapies, or probiotic use) or overlap with other gastrointestinal disorders. An important selection bias was observed in some studies, where patients with IF underwent testing for SIBO after receiving empiric antibiotic therapy for SIBO.

One of the important factors contributing to the clinical heterogeneity is the widely recognized limitation of the available tests for diagnosing SIBO, as they are suboptimal in relation to sensitivity and specificity.^32^ Quantitative microbial culture of small bowel aspirate (direct) has been considered as gold standard for diagnosing SIBO. However, these have several limitations like being invasive, require an endoscopy with specialized equipment, prone to cross‐contamination by oropharyngeal microbes and luminal secretions, and there is lack of consensus on the site of sampling in the small intestine and reliable thresholds of bacterial counts for diagnosing SIBO.^10^ The direct tests have been replaced by breath tests (indirect test) in clinical setting for diagnosing SIBO. However, the breath tests are not adequately standardized and therefore have also significant methodological limitations and lack sensitivity and specificity for SIBO diagnosis.^33^ It should be added that in the current meta‐analysis the majority of studies were conducted in a pediatric population and the diagnosis of SIBO was based on clinical symptoms suspicious for SIBO rather than clinically available diagnostic tests, which further reduces the accuracy of the diagnosis in these cases. Furthermore, it was not possible to extract data for conducting subgroup analysis based on the type of diagnostic test used to identify SIBO in patients with IF.

For those studies that used small bowel aspirates for culture‐based tests of SIBO positivity in IF patients, the most commonly reported bacteria include both Gram‐positive (*Streptococcus viridans* and *Enterococcus* species) and Gram‐negative ( *Escherichia coli* and *Klebsiella pneumoniae* ) taxa. While many members of all these taxa are recognized for their capacity to grow in the microaerophilic conditions, they are most likely prevalent within the gut lumen during PN administration. However, the resident microbiota throughout the GI tract during periods of PN are also most likely to favor other unidentified taxa with the capacity to utilize and release nutrients from mucins, sloughed epithelial cells and other host‐derived secretions. As such, whether the bacterial taxa reported in these studies examined here are the cause or consequence of SIBO in IF remains unclear. These are aligned with the findings of recent study by Leite *et al*.^11^ that confirmed that few specific *E*. *coli* and *Klebsiella* strains/species appear responsible for the majority of overgrowth and SIBO symptoms. None of the studies included in this meta‐analysis measured methane positivity during breath testing. The significance of breath methane measurements in individuals suspected of having intestinal dysbiosis is suggested by the guidelines of the American College of Gastroenterology for SIBO.^34^

One of the key findings of this systematic review and meta‐analysis is the sixfold increased risk of SIBO in patients with IF on PN (OR = 6.0, 95% CI 3.0–11.9, *P* = 0.0001). The association between SIBO and PN dependence is likely multifactorial. SIBO may cause structural changes such as atrophy of small intestinal villi^9^ with subsequent alterations of small intestinal absorption. Thus, patients with SIBO are at risk for increased intestinal malabsorption and the resultant need for PN support. Furthermore, patients with SIBO may experience limited tolerance to enteral nutrition due to symptoms related to intestinal malabsorption, further necessitating the use of PN. This would potentially explain why patients with IF and positive for SIBO require PN for a longer duration as compared with those without SIBO (34.5 ± 41.5 months *vs* 13.9 ± 12.6 months *P* = 0.097). The administration of PN requires a central venous catheter and hence increases the risk for CRBSI in patients with IF. Furthermore, SIBO has been linked to a higher incidence of CRBSI via bacterial translocation, through increased intestinal permeability seen in patients with IF. Although limited by the small number of studies, we found no link in the prevalence of CRBSI in patients with IF and SIBO.

SBS is the most common cause of IF in both adults and children.^3^ The pooled prevalence rate of SIBO in 188 patients with IF due to SBS was 49.0% (95% CI 33.8–64.5). However, data could not be extracted to conduct subgroup analysis to compare prevalence of SIBO in IF patients due to SBS and non‐SBS. PPI use has been considered as a risk factor for SIBO in various gastrointestinal conditions.^35^ Chronic acid suppression and the resultant hypochlorhydria associated with PPI use has been suggested to alter the intraluminal environment to promote the growth of colonic bacteria in the small intestine.^36^ However, a recent study^37^ showed that SIBO rates were not significantly different between subjects on a PPI as compared with those not on a PPI, based upon duodenal aspirate and culture (> 10^3^ CFU/mL) or 16S sequencing. Thus, the link between SIBO and PPI use remains controversial. In the current systematic review although limited by a small sample size, SIBO prevalence was proportionally (but not significantly) higher in IF patients on an acid‐suppressing agent as compared with those not on an acid‐suppressing agent (72.0% [95% CI 57.5–83.8] *vs* 47.6%, [95% CI 25.7–70.2]). Thus, the true link between SIBO in IF and PPI use remains to be explored. Patients with IF often require long‐term and cyclical use of antibiotics to manage their symptoms. Antibiotics are commonly prescribed based on clinical suspicion of SIBO, without relying on specific diagnostic tests. Despite this, only two out of the nine studies included in this meta‐analysis reported on transient improvement in gastrointestinal symptoms after antibiotic therapy using a variety of broad‐spectrum antibiotics; thus, a definitive conclusion on the most effective antibiotic for treating SIBO positive patients with IF cannot be drawn. Despite this, only two studies included in this meta‐analysis reported on transient improvement in gastrointestinal symptoms in all SIBO positive patients with IF. Moreover, they did not provide information regarding the normalization of SIBO diagnostic tests after antibiotic treatment. It is important that the risks of long‐term cyclical antibiotic therapy including antibiotic resistance, agent specific adverse effects, increased cost, and *Clostridium difficile* colitis should be appropriately investigated to help minimize antimicrobial resistance in this medically complex population.

Patients with IF often have abnormal intestinal anatomy, which can predispose to SIBO. SIBO is believed to occur as a consequence of intestinal dilation and subsequent stasis, which in turn promotes more bacterial proliferation and inflammation.^14^ While the small sample size and limited number of studies hindered a comprehensive subgroup analysis, the absence of an ICV was identified as a potential risk factor for SIBO in patients with IF (OR = 1.7, 95% CI 0.8–3.6, *P* = 0.174). Similarly, a shorter residual small bowel length (but not the colonic length) was also identified as a potential risk factor for SIBO in patients with IF. However, like the previous findings, this association did not reach statistical significance.

SIBO is now considered a cause or contributor for symptoms in patients with highly prevalent conditions such as DGBI,^38^, ^39^ celiac disease,^40^ or IBD.^41^ Symptoms linked with SIBO often overlap with other gastrointestinal conditions hence are considered as poor predictors of bacterial overgrowth. Grace *et al*.^42^ found that diarrhea was the most prevalent symptom associated with SIBO, followed by abdominal pain and bloating. However, no study explored the symptom severity in patients with IF and the association with SIBO. Across various studies included in this meta‐analysis, we were unable to extract data and conduct subgroup analysis to identify a specific symptom or cluster of symptoms that could reliably predicts the SIBO in IF. This meta‐analysis also aimed to assess various risk factors for SIBO in patients with IF, but no effect sizes or ORs could be calculated regarding the prevalence of SIBO and the markers of inflammation such as fecal calprotectin and intestinal permeability or duration of PN. This information has been outlined in a descriptive fashion as the available studies did not allow data extraction to perform the appropriate statistical analyses.

There are limitations of this systematic review and meta‐analysis. The case–control studies included patients with a variety of diseases or unexplained gastrointestinal symptoms as controls, and moreover, none of the controls underwent assessment for SIBO. Including only cases from case–control studies in this meta‐analysis certainly has some limitations. Firstly, the lack of a control group for comparison hinders the assessment of association and causality. Without controls, the generalizability of findings is affected, as it does not represent the broader population.^43^ In addition, accounting for confounding factors becomes challenging. Thus, the absence of controls limits the interpretation and comprehensive understanding of the exposure–outcome relationship). In addition, small sample sizes (e.g., < 50 subjects per group) in majority of the studies (six of the nine studies) limited the statistical power of some of the sub‐group analyses.

In summary, this is the first systematic review and meta‐analyses, which reveals an increased prevalence of SIBO in patients with IF. PN dependence appeared to be a risk factor for SIBO in IF. Although limited by the small sample size (and the small number of studies), anatomical, and structural changes such as absence of an ICV, shorter residual small bowel (but not colon), acid‐suppressing agents, and longer duration of PN appeared to be possible risk factors for SIBO in patients with IF and need further investigation. While only limited data were available, we did not find any significant difference in SIBO prevalence according to etiology of IF.

The findings of this meta‐analysis must be interpreted with caution. The quality of evidence is low, and this can be attributed mainly to substantial clinical heterogeneity seen in the studies included in this meta‐analysis. In order to facilitate meaningful clinical investigations in the context of the relative rarity of IF, it is essential that centers involved in IF management collaborate and establish a joint clinical database. Such a collaborative effort will enable a more comprehensive and impactful approach to conducting research in this field.

## Supporting information


**Figure S1:** Search strategy for MEDLINE.
**Figure S2:** Forest plot of studies showing prevalence of SIBO in patients with IF, on parenteral nutrition (PN) 74.9% (95%CI 59.8–85.6, *P* = 0.002), (I_2_ = 35.5, *P* = 0.157).
**Figure S3:** Forest plot of studies showing prevalence of SIBO in patients with IF, due to short bowel syndrome (SBS) 49.0% (95%CI 33.8–64.5, *P* = 0.904), (I_2_ = 73.4, *P* = 0.005).
**Figure S4:** Forest plot of studies showing prevalence of SIBO in patients with IF, including only high‐quality studies 66.1% (95%CI 54.9–75.8, *P* = 0.006), (I_2_ = 58.2, *P* = 0.067).
**Figure S5:** Forest plot of studies showing prevalence of SIBO in patients with IF, without an intact ileo‐cecal valve (ICV) compared to those with an ICV (OR = 1.7, 95%CI 0.8–3.6, *P* = 0.174), (I_2_ = 10.0, *P* = 0.343).
**Table S1:** Eligibility criteria for the studies included in systematic review and meta‐analysis.
**Table S2:** Assessment of risk factors for SIBO in patients with IF in the studies included in the systematic review and meta‐analysis.
**Table S3:** Assessment of cut off criteria for diagnosing SIBO in patients with IF.
**Table S4:** Joanna Briggs Institute (JBI) Critical Appraisal Tools for assessment of quality of cohort studies and the case groups of the case–control studies included in the systematic review and meta‐analysis.
**Table S5:** Studies assessing the effect of proton pump inhibitor (PPI) on small intestinal bacterial overgrowth (SIBO) prevalence in patients with intestinal failure (IF).
**Table S6:** Composition of small bowel aspirate in patients with intestinal failure (IF) diagnosed with small intestinal bacterial overgrowth (SIBO), using a cut‐off threshold of 10^5^ colony forming units/milliliter.
**Table S7:** Predominant gastrointestinal symptoms in patients with intestinal failure (IF) with small intestinal bacterial overgrowth (SIBO).
**Table S8:** Studies assessing the prevalence of small intestinal bacterial overgrowth (SIBO) in patients with intestinal failure (IF) according to anatomy.
**Table S9:** Studies assessing the prevalence of small intestinal bacterial overgrowth (SIBO) in patients with intestinal failure (IF) according to anatomy.
**Table S10:** Studies assessing the prevalence of small intestinal bacterial overgrowth (SIBO) in patients with intestinal failure (IF) according to anatomy and outcomes.

## Data Availability

Aggregate, rather than individual‐level, data were included in these analyses from published manuscripts and conference publications, which are publicly available.
