# Supplementary material for: Prevalence of small intestinal bacterial overgrowth in intestinal failure syndrome: A systematic review and meta‐analysis
Source: J Gastroenterol Hepatol. 2024 Jun 27;39(11):2308–18. doi: 10.1111/jgh.16668 (PMC11618250; doi:10.1111/jgh.16668)
Supplement: Supplementary file 1 — Figure S1: Search strategy for MEDLINE. Figure S2: Forest plot of studies showing prevalence of SIBO in patients with IF, on parenteral nutrition (PN) 74.9% (95%CI 59.8–85.6, P = 0.002), (I2 = 35.5, P = 0.157). Figure S3: Forest plot of studies showing prevalence of SIBO in patients with IF, due to short bowel syndrome (SBS) 49.0% (95%CI 33.8–64.5, P = 0.904), (I2 = 73.4, P = 0.005). Figure S4: Forest plot of studies showing prevalence of SIBO in patients with IF, including only high‐quality studies 66.1% (95%CI 54.9–75.8, P = 0.006), (I2 = 58.2, P = 0.067). Figure S5: Forest plot of studies showing prevalence of SIBO in patients with IF, without an intact ileo‐cecal valve (ICV) compared to those with an ICV (OR = 1.7, 95%CI 0.8–3.6, P = 0.174), (I2 = 10.0, P = 0.343). Table S1: Eligibility criteria for the studies included in systematic review and meta‐analysis. Table S2: Assessment of risk factors for SIBO in patients with IF in the studies included in the systematic review and meta‐analysis. Table S3: Assessment of cut off criteria for diagnosing SIBO in patients with IF. Table S4: Joanna Briggs Institute (JBI) Critical Appraisal Tools for assessment of quality of cohort studies and the case groups of the case–control studies included in the systematic review and meta‐analysis. Table S5: Studies assessing the effect of proton pump inhibitor (PPI) on small intestinal bacterial overgrowth (SIBO) prevalence in patients with intestinal failure (IF). Table S6: Composition of small bowel aspirate in patients with intestinal failure (IF) diagnosed with small intestinal bacterial overgrowth (SIBO), using a cut‐off threshold of 105 colony forming units/milliliter. Table S7: Predominant gastrointestinal symptoms in patients with intestinal failure (IF) with small intestinal bacterial overgrowth (SIBO). Table S8: Studies assessing the prevalence of small intestinal bacterial overgrowth (SIBO) in patients with intestinal failure (IF) according to anatomy. Table S9: Studies a [file JGH-39-2308-s001.docx]

**Supplementary material:**

**Legend of Figures:**

**Figure S1:** Search strategy for MEDLINE**.**

**Figure S2:** Forest plot of studies showing prevalence of SIBO in patients with IF, on parenteral nutrition (PN) 74.9% (95%CI 59.8-85.6, p=0.002), (I_2_=35.5, p=0.157).

**Figure S3:** Forest plot of studies showing prevalence of SIBO in patients with IF, due to short bowel syndrome (SBS) 49.0% (95%CI 33.8-64.5, p=0.904), (I_2_=73.4, p=0.005).

**Figure S4:** Forest plot of studies showing prevalence of SIBO in patients with IF, including only high-quality studies 66.1% (95%CI 54.9-75.8, p=0.006), (I_2_=58.2, p=0.067).

**Figure S5:** Forest plot of studies showing prevalence of SIBO in patients with IF, without an intact ileo-cecal valve (ICV) compared to those with an ICV (OR=1.7, 95%CI 0.8-3.6, p=0.174), (I_2_=10.0, p=0.343).

**Legend of Tables:**

**Table S1:** Eligibility criteria for the studies included in systematic review and meta-analysis.

**Table S2:** Assessment of risk factors for SIBO in patients with IF in the studies included in the systematic review and meta-analysis.

**Table S3:** Assessment of cut off criteria for diagnosing SIBO in patients with IF.

**Table S4:**  Joanna Briggs Institute (JBI) Critical Appraisal Tools for assessment of quality of cohort studies and the case groups of the case-control studies included in the systematic review and meta-analysis.

**Table S5:** Studies assessing the effect of proton pump inhibitor (PPI) on small intestinal bacterial overgrowth (SIBO) prevalence in patients with intestinal failure (IF).

**Table S6:** Studies evaluating the effect of antibiotic treatment in patients with intestinal failure (IF) and small intestinal bacterial overgrowth (SIBO).

**Table S7:** Composition of small bowel aspirate in patients with intestinal failure (IF) diagnosed with small intestinal bacterial overgrowth (SIBO), using a cut-off threshold of 10^5^ colony forming units/millilitre.

**Table S8:** Studies assessing the prevalence of small intestinal bacterial overgrowth (SIBO) in patients with intestinal failure (IF) according to anatomy and outcomes.

| **Database search strategy MEDLINE(PubMed)** |
| --- |
| #1. "short bowel syndrome"[tiab] OR "short gut syndrome"[tiab] OR "short gut"[tiab] OR "SBS"[ti] OR short bowel syndrome [mh] OR "intestinal failure"[tiab] OR "intestine failure"[tiab]  #2. "small intestinal bacterial overgrowth"[tiab] OR "SIBO"[ti] OR "bacterial overgrowth"[tiab] #3. #1 AND #2  #4. "small bowel"[tiab:~2] OR "small intestine"[tiab:~2] OR "small intestinal"[tiab:~2] OR intestine, small[mh]  #5. "bacterial overgrowth"[tiab: ~2] OR "bacteria overgrowth"[tiab: ~2] OR "dysbiosis"[tiab] OR microbiome[tiab] OR microbiota[tiab] OR flora[tiab] OR probiotic*[tiab] OR prebiotic*[tiab] OR symbiotic*[tiab] OR "breath tests"[tiab: ~2] OR "breath test"[tiab~2] OR "SIBO"[tiab] OR gastrointestinal microbiome[mh] OR breath tests[mh] OR "blind loop syndrome"[tiab] OR "blind loop syndrome"[mh]  #6. #4 AND #5  #7. "short bowel"[tiab:~2] OR "short small bowel"[tiab:~2]  #8. #7 AND syndrome[tiab]  #9. short bowel syndrome[mh] OR "short bowel length"[tiab:~2] OR "bowel resection"[tiab:~2] OR "bowel resections"[tiab:~2] OR intestinal failure[mh] OR "intestinal failure"[tiab:~2] OR "intestinal failures"[tiab:~2]  #10. #8 OR #9  #11. #6 AND #10  #12. #3 OR #11 |

**Figure S1:** Search strategy for MEDLINE**.**

**
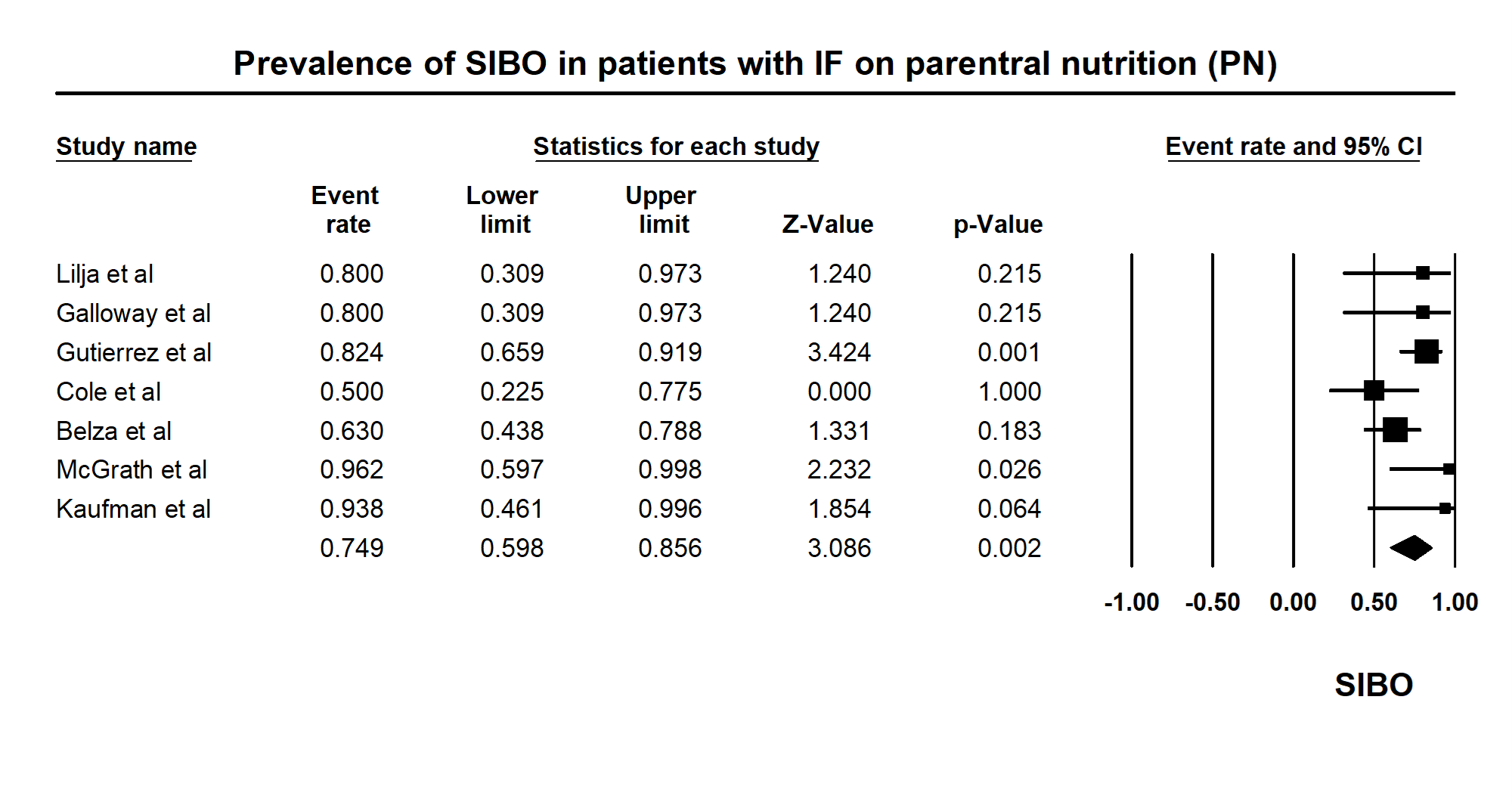
**

**Figure S2:** Forest plot of studies showing prevalence of SIBO in patients with IF, on parenteral nutrition (PN) 74.9% (95%CI 59.8-85.6), (I_2_=35.5, p=0.157).

**Figure S3:** Forest plot of studies showing prevalence of SIBO in patients with IF, due to short bowel syndrome (SBS), 49.0% (95%CI 33.8-64.5), (I_2_=73.4, p=0.005).

**
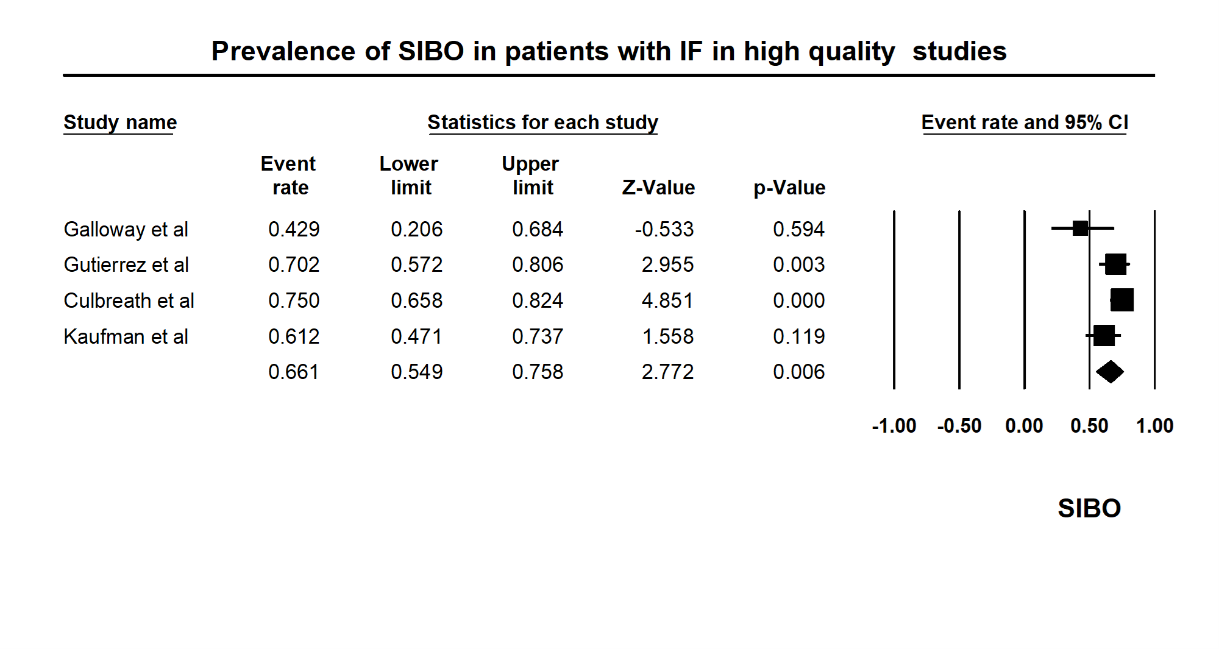
**

**Figure S4:** Forest plot of studies showing prevalence of SIBO in patients with IF, including only high-quality studies 66.1% (95%CI 54.9-75.8), (I_2_=58.2, p=0.067).

**Figure S5:** Forest plot of studies showing prevalence of SIBO in patients with IF, without an intact ileo-cecal valve (ICV) compared to those with an ICV (OR=1.7, 95%CI 0.8-3.6, p=0.174), (I_2_=10.0, p=0.343).

**Table S1:** Eligibility criteria for the studies included in systematic review and meta-analysis.

| **Eligibility criteria** |
| --- |
| - Case-control, prevalence or cohort studies published as full papers in peer reviewed journals or conference abstracts. |
| - Patients with an established diagnosis of intestinal failure and/or short gut syndrome/short bowel syndrome. - Control group, referred to as ‘controls’ included ‘healthy asymptomatic controls’ as well as ‘patient controls’ including patients undergoing evaluation for unexplained gastrointestinal ‘syndromes’ (e.g., anemia, dysphagia, diarrhea etc.). - Studies reporting on efficacy data after antibiotic treatment of small intestinal bacterial overgrowth (SIBO) in patients with intestinal failure were also included |
| - Participants not specially selected. |

**Table S2:** Assessment of risk factors for SIBO in patients with IF in the studies included in the systematic review and meta-analysis.

| **No** | **Author** | **Prior antibiotic use** | **Concurrent PPI use** | **Treatment with antibiotic** | **Duration of treatment** | **Treatment efficacy** |
| --- | --- | --- | --- | --- | --- | --- |
| 1 | Lilja et al^1^ | NA | NA | Oral metronidazole, trimethoprim-sulfamethoxazole, gentamicin, or amoxicillin-clavulanic acid | Variable duration | NA |
| 2 | Galloway et al^2^ | None within prior 2 weeks | 6/14 | NA | NA | NA |
| 3 | Gutierrez et al^3^ | Yes | 44/57 | All children had been initially treated with empiric antibiotic. | NA | NA |
| 4 | Cole et al^4^ | None within prior 2 weeks | NA | NA | NA | NA |
| 5 | Belza et al^5^ | NA | 31/55 | most prescribed antibiotics were metronidazole (42.9%), ciprofloxacin (21.4%), and gentamicin (12.5%) | A typical regimen would be twice daily for 7 days/ month on a repeating cycle | Six months after endoscopy and antibiotic therapy, statistically significant improvements in emesis & feeding intolerance (58.9% to 23.2%, P < 0.001), abdominal pain (16.1% to 7.1%, P = 0.02), high stool output (42.9% to 19.6%, P < 0.01), and GI bleeding (19.6% to 3.6%, P < 0.01). BMI-for-age z scores improved significantly (−0.03±0.94 vs 0.27±0.82, P = 0.03), and weight-for-age z scores trended toward improvement but was not statistically significant (−0.86±0.97 to −0.65±0.96, P = 0.05). |
| 6 | Culbreath et al^6^ | Yes | NA | NA | NA | NA |
| 7 | McGrath et al^7^ | NA | NA | NA | NA | NA |
| 8 | Dibaise et al^8^ | NA | NA | NA | NA | NA |
| 9 | Kaufman et al^9^ |  |  | Oral gentamicin, metronidazole, cephalexin, and trimethoprim-sulfamethoxazole | 1 to 2 weeks | Reacquisition of enteral feeding tolerance and transient reduction in diarrhea, distention, and flatulence. |

FD: functional dyspepsia; SIBO: small intestinal bacterial overgrowth; GBT: glucose breath test; NA: not applicable.

**Table S3:** Assessment of cut off criteria for diagnosing SIBO in patients with IF.

| **No** | **Author** | **Mode of diagnosis of SIBO** | **Type of breath collection devices** | **Dose of substrate** | **Cut off criteria for SIBO diagnosis** |
| --- | --- | --- | --- | --- | --- |
| 1 | Lilja et al^1^ | NA | NA | NA | NA |
| 2 | Galloway et al^2^ | Duodenal aspirate and culture | NA | NA | >10^5^ colony forming units per milliliter (CFU/mL) |
| 3 | Gutierrez et al^3^ | Duodenal aspirate and culture | NA | NA | >10^5^ CFU/mL |
| 4 | Cole et al^4^ | GHBT | Microlyzer (Model SC, QuinTron Instrument Co. Milwaukee, WI). | 1g/kg D-glucose solution | Baseline hydrogen value ≥20 parts per million (ppm) or increase from baseline of ≥ 10 ppm after the ingestion of glucose. |
| 5 | Belza et al^5^ | Clinical symptoms | NA | NA | NA |
| 6 | Culbreath et al^6^ | Duodenal aspirate and culture | NA | NA | >10^5^ CFU/mL |
| 7 | McGrath et al^7^ | Small bowel aspirate and culture OR Hydrogen breath test | NA | NA | NA |
| 8 | Dibaise et al^8^ | Duodenal aspirate and culture  OR  GHBT | NA | NA | >10^5^ CFU/mL  Baseline hydrogen value >20 ppm or increase from baseline of > 20 ppm <60 min after ingestion. |
| 9 | Kaufman et al^9^ | Duodenojejunal aspirate and culture | NA | NA | Elevated counts |

IF: intestinal failure; SIBO: small intestinal bacterial overgrowth; GHBT: glucose hydrogen breath test; NA: not applicable.

**Table S4:**  Joanna Briggs Institute (JBI) Critical Appraisal Tools for assessment of quality of cohort studies and the case groups of the case-control studies included in the systematic review and meta-analysis.

|  | **1. Was the sample frame appropriate to address the target population?** | **2. Were study participants sampled in an appropriate way?** | **3. Was the sample size adequate?** | **4. Were the study subjects and the setting described in detail?** | **5. Was the data analysis conducted with sufficient coverage of the identified sample?** | **6. Were valid methods used for the identification of the condition?** | **7. Was the condition measured in a standard, reliable way for all participants?** | **8. Was there appropriate statistical analysis?** | **9. Was the response rate adequate, and if not, was the low response rate managed appropriately?** | **Risk of bias** |
| --- | --- | --- | --- | --- | --- | --- | --- | --- | --- | --- |
| Lilja et al^1^ | Yes | No | No | Yes | No | No | Yes | Yes | No | High |
| Galloway et al^2^ | Yes | Yes | Yes | Yes | Yes | Yes | Yes | Yes | Yes | Low |
| Gutierrez et al^3^ | Yes | Yes | Yes | Yes | NA | Yes | Yes | Yes | NA | Low |
| Cole et al^4^ | No | No | No | Yes | No | Yes | Yes | Yes | NA | High |
| Belza et al^5^ | Yes | No | Yes | No | Yes | No | No | Yes | Yes | Moderate |
| Culbreath et al^6^ | No | Yes | Yes | Yes | Yes | Yes | Yes | Yes | Yes | Low |
| McGrath et al^7^ | Yes | Yes | No | Yes | NA | No | Yes | NA | NA | High |
| Dibaise et al^8^ | Yes | NA | Yes | No | NA | Yes | Yes | NA | NA | High |
| Kaufman et al^9^ | Yes | Yes | Yes | Yes | Yes | Yes | Yes | Yes | NA | Low |

**Table S5:** Studies assessing the effect of proton pump inhibitor (PPI) on small intestinal bacterial overgrowth (SIBO) prevalence in patients with intestinal failure (IF).

| **No** | **Author** | **Patients with IF,**  **n** | **SIBO in patients with IF,**  **n (%)** | **Patients with IF on PPI,**  **n** | **SIBO in patients with IF on PPI,**  **n (%)** | **Patients with IF not on PPI,**  **n** | **SIBO in patients with IF not on PPI,**  **n (%)** |
| --- | --- | --- | --- | --- | --- | --- | --- |
| 1 | Galloway et al^2^ | 14 | 6 (42.9) | 6 | 3 (50.0) | 8 | 3 (37.5) |
| 2 | Gutierrez et al^3^ | 57 | 40 (70.2) | 44 | 33 (75.0) | 13 | 7 (53.8) |
| 3 | Culbreath et al^6^ | 104 | 78 (75.0) | NA | 31 (NA) | NA | 24 (NA) |

NA: not applicable; n: number.

**Table S6:** Composition of small bowel aspirate in patients with intestinal failure (IF) diagnosed with small intestinal bacterial overgrowth (SIBO), using a cut-off threshold of 10^5^ colony forming units/millilitre.

| **No** | **Author** | **Type of microbes isolated in small bowel aspirate** |
| --- | --- | --- |
| 1 | Lilja et al^1^ | Utilizing 16SrRNA sequencing (on fecal samples), the Shannon diversity index was significantly reduced in children with SBS on PN compared to children weaned from PN, with a predominance of *Enterobacteriacae* in majority of children on SBS while on PN. In SBS patients, all off PN, there was a more diverse microbiota composition and a more uniform distribution of taxonomic families. However, the microbial diversity was reduced as compared to that in controls. |
| 2 | Galloway et al^2^ | *Lactobacillus*, *Bifidobacterium*, ***Enterococcus***, *Streptococcus*, *Gemella,* *Veillonella*, *Morganella*, ***Klebsiella*,** *Escherichia.* |
| 3 | Gutierrez et al^3^ | ***Streptococci viridian, Enterococcus****, Lactobacillu, Rothia mucilaginosa, Enterococcus faecium, Enterococcus faecalis, Corynebacterium, Stomatococcus,* ***Escherichia coli, Klebsiella pneumoniae****, Pseudomonas aeruginosa, Enterobacter cloacae, Klebsiella oxytoca, Nonpathogenic neisseria, Haemophilus influenzae, Haemophilus, Klebsiella ozonae, Proteus mirabilis, Citrobacter diversus, Citrobacter freundii, Stenotrophomonas maltophilia, Morganella morganii, Candida.*  ***The most common organisms associated with a CRBSI were Staphylococcus (non-aureus), Escherichia coli, and Klebsiella pneumoniae.*** |
| 4 | Cole et al^4^ | NA |
| 5 | Belza et al^5^ | NA |
| 6 | Culbreath et al^6^ | ***Streptococci viridans, Lactobacillus, Enterococcus,*** *Staphylococcu (S. aureus), Rothia mucilaginosa, Bifidobacterium, Actinomyces, Clostridium Peptostreptococcus,* ***Escherichia coli, Klebsiella, Haemophilus****, Enterobacter, Citrobacter, Pseudomonas aeruginosa, Neisseria, Morganella morganii, Bacteroides, Serratia, Fusobacterium, Eikenella corrodens, Pantoea, Proteus, Candida albicans.* |
| 7 | McGrath et al^7^ | NA |
| 8 | Dibaise et al^8^ | 19 of 28 duodenal aspirates showed excessive growth of either aerobes only (10 of 19), or both aerobes and anaerobes (9 of 19). |
| 9 | Kaufman et al^9^ | NA |

***Prominent genera/species cultured among all aspirates highlighted in bold.*** NA: not available.

**Table S7:** Predominant gastrointestinal symptoms in patients with intestinal failure (IF) with small intestinal bacterial overgrowth (SIBO).

| No | Author | Patients with IF,  n | SIBO in patients with IF,  n (%) | Prominent symptoms associated with SIBO |
| --- | --- | --- | --- | --- |
| 1 | Lilja et al^1^ | 11 | 4 (36.4) | NA |
| 2 | Galloway et al^2^ | 14 | 6 (42.9) | NA |
| 3 | Gutierrez et al^3^ | 57 | 40 (70.2) |  |
| 4 | Cole et al^4^ | 10 | 5 (50) | NA |
| 5 | Belza et al^5^ | 102 | 35 (34.3) | NA |
| 6 | Culbreath et al^6^ | 104 | 78 (75.0) | emesis and feeding intolerance, high stool output, and abdominal distention as the predominant symptoms associated with SIBO in patients with IF |
| 7 | McGrath et al^7^ | 17 | 12 (70.6) | diarrhea being the most reported symptom, followed by vomiting/nausea, bloating, abdominal pain, and feed intolerance |
| 8 | Dibaise et al^8^ | 43 | 27 (62.8) | NA |
| 9 | Kaufman et al^9^ | 49 | 30 (61.2) | NA |

NA: not applicable; n: number.

**Table S8:** Studies assessing the prevalence of small intestinal bacterial overgrowth (SIBO) in patients with intestinal failure (IF) according to anatomy.

| No | Author | Patients with IF,  n | SIBO in patients with IF,  n (%) | Patients with IF with an ICV,  n | SIBO in patients with IF with an ICV,  n (%) | Patients with IF without an ICV,  n | SIBO in patients with IF without an ICV,  n (%) |
| --- | --- | --- | --- | --- | --- | --- | --- |
| 1 | Lilja et al^1^ | 11 | 4 (36.4) | 4 | 0 (0) | 7 | 4 (57.1) |
| 2 | Galloway et al^2^ | 14 | 6 (42.9) | 3 | 1 (33.3) | 11 | 5 (45.5) |
| 3 | Gutierrez et al^3^ | 57 | 40 (70.2) | 16 | 12 (75.0) | 41 | 28 (68.0) |
| 4 | Cole et al^4^ | 10 | 5 (50) | NA | 2 | NA | 3 |
| 5 | Belza et al^5^ | 102 | 35 (34.3) | 62 | 17 (48.6) | 40 | 18 (45.0) |
| 6 | Culbreath et al^6^ | 104 | 78 (75.0) | NA | 18 (NA) | NA | 33 (NA) |
| 7 | McGrath et al^7^ | 17 | 12 (70.6) | NA | NA | NA | NA |
| 8 | Dibaise et al^8^ | 43 | 27 (62.8) | NA | NA | NA | NA |
| 9 | Kaufman et al^9^ | 49 | 30 (61.2) | NA | NA | NA | NA |

NA: not applicable; n: number; ICV: ileo-cecal valve.

**Table S9:** Studies assessing the prevalence of small intestinal bacterial overgrowth (SIBO) in patients with intestinal failure (IF) according to anatomy.

| No | Author | Patients with IF,  n | SIBO in patients with IF,  n (%) | Length of remnant SB in Patients with IF,  Mean (SD), cm | Length of remnant SB in Patients with IF with SIBO, Mean (SD),  cm | Length of remnant SB in Patients with IF without SIBO, Mean (SD),  cm | Percentage of remnant SB in Patients with IF, Mean (SD) | Percentage of remnant SB in Patients with IF with SIBO, Mean (SD) | Percentage of remnant SB in Patients with IF without SIBO, Mean (SD) |
| --- | --- | --- | --- | --- | --- | --- | --- | --- | --- |
| 1 | Lilja et al^1^ | 11 | 4 (36.4) | NA | Jejunum 13.75 (4.8) | NA | NA | NA | NA |
| 2 | Galloway et al^2^ | 14 | 6 (42.9) | NA | NA | NA | 43.8 (29.9) | 27.8 (17.9) | 55.8 (32.4) |
| 3 | Gutierrez et al^3^ | 57 | 40 (70.2) | NA | NA | NA | NA | NA | NA |
| 4 | Cole et al^4^ | 10 | 5 (50) | 43.2 (13.5) | 39.8 (13.0) | 46.6 (14.3) | 20.9 (5.8) | 20.8 (6.8) | 21 (5.4) |
| 5 | Belza et al^5^ | 102 | 35 (34.3) | 90.0 (NA) | 81.7 (NA) | 94.3 (NA) | 59.2 (NA) | 45.4 (NA) | 66.5 (NA) |
| 6 | Culbreath et al^6^ | 104 | 78 (75.0) | NA | NA | NA | NA | 23.4 (15.7) | NA |
| 7 | McGrath et al^7^ | 17 | 12 (70.6) | NA | NA | NA | NA | NA | NA |
| 8 | Dibaise et al^8^ | 43 | 27 (62.8) | NA | NA | NA | NA | NA | NA |
| 9 | Kaufman et al^9^ | 49 | 30 (61.2) | NA | NA | NA | NA | NA | NA |

NA: not applicable; n: number; SB: small bowel; SD: standard deviation.

**Table S10:** Studies assessing the prevalence of small intestinal bacterial overgrowth (SIBO) in patients with intestinal failure (IF) according to anatomy and outcomes.

| No | Author | Patients with IF,  n | SIBO in patients with IF,  n (%) | Length of remnant colon in Patients with IF,  Mean (SD), cm | Length of remnant colon in Patients with IF with SIBO, Mean (SD),  cm | Length of remnant colon in Patients with IF without SIBO, Mean (SD),  cm | Percentage of remnant colon in Patients with IF, Mean (SD) | Percentage of remnant colon in Patients with IF with SIBO, Mean (SD) | Percentage of remnant colon in Patients with IF without SIBO, Mean (SD) |
| --- | --- | --- | --- | --- | --- | --- | --- | --- | --- |
| 1 | Lilja et al^1^ | 11 | 4 (36.4) | NA | NA | NA | 68.2 (26.3) | 45.8 (8.4) | 80.9 (24.4) |
| 2 | Galloway et al^2^ | 14 | 6 (42.9) | NA | NA | NA | NA | NA | NA |
| 3 | Gutierrez et al^3^ | 57 | 40 (70.2) | NA | NA | NA | NA | NA | NA |
| 4 | Cole et al^4^ | 10 | 5 (50) | NA | NA | NA | 73 (25.0) | 76 (25.1) | 70 (27.4) |
| 5 | Belza et al^5^ | 102 | 35 (34.3) | 22.6 (NA) | 29.0 | 19.2 (NA) | 76.5 (NA) | 68.9 (NA) | 80.4 (NA) |
| 6 | Culbreath et al^6^ | 104 | 78 (75.0) | NA | NA | NA | NA | NA | NA |
| 7 | McGrath et al^7^ | 17 | 12 (70.6) | NA | NA | NA | NA | NA | NA |
| 8 | Dibaise et al^8^ | 43 | 27 (62.8) | NA | NA | NA | NA | NA | NA |
| 9 | Kaufman et al^9^ | 49 | 30 (61.2) | NA | NA | NA | NA | NA | NA |

NA: not applicable; n: number; SD: standard deviation.

**References:**

1. Engstrand Lilja H, Wefer H, Nyström N, et al. Intestinal dysbiosis in children with short bowel syndrome is associated with impaired outcome. Microbiome 2015;3:18.

2. Galloway D, Mezoff E, Zhang W, et al. Serum Unconjugated Bile Acids and Small Bowel Bacterial Overgrowth in Pediatric Intestinal Failure: A Pilot Study. JPEN J Parenter Enteral Nutr 2019;43:263-270.

3. Gutierrez IM, Kang KH, Calvert CE, et al. Risk factors for small bowel bacterial overgrowth and diagnostic yield of duodenal aspirates in children with intestinal failure: a retrospective review. J Pediatr Surg 2012;47:1150-4.

4. Cole CR, Frem JC, Schmotzer B, et al. The rate of bloodstream infection is high in infants with short bowel syndrome: relationship with small bowel bacterial overgrowth, enteral feeding, and inflammatory and immune responses. J Pediatr 2010;156:941-947.e1.

5. Belza C, Betts Z, de Silva N, et al. Factors Related to the Development of Small-Bowel Bacterial Overgrowth in Pediatric Intestinal Failure: A Retrospective Cohort Study. JPEN J Parenter Enteral Nutr 2020;44:1280-1284.

6. Culbreath K, Knell J, Keefe G, et al. Antibiotic Therapy for Culture-Proven Bacterial Overgrowth in Children With Intestinal Failure Results in Improved Symptoms and Growth. J Pediatr Gastroenterol Nutr 2022;75:345-350.

7. McGrath KH, Pitt J, Bines JE. Small intestinal bacterial overgrowth in children with intestinal failure on home parenteral nutrition. JGH Open 2019;3:394-399.

8. Dibaise JK, Young RJ, Vanderhoof JA. Enteric microbial flora, bacterial overgrowth, and short-bowel syndrome. Clin Gastroenterol Hepatol 2006;4:11-20.

9. Kaufman SS, Loseke CA, Lupo JV, et al. Influence of bacterial overgrowth and intestinal inflammation on duration of parenteral nutrition in children with short bowel syndrome. J Pediatr 1997;131:356-61.
